# Supplementary material for: The Development and Validation of the “Hippocratic Hypertension Self-Care Scale”
Source: Healthcare (Basel). 2023 Sep 18;11(18):2579. doi: 10.3390/healthcare11182579 (PMC10530843; doi:10.3390/healthcare11182579)
Supplement: Supplementary file 1 [file healthcare-11-02579-s001.zip › healthcare-2493246-supplementary.pdf]

Supplementary Materials:

Table S1: Hippocratic hypertension self-care scale

Please, read carefully the following sentences and choose the most appropriate answer, based on your self-behavior regarding medication, diet, exercise, lifestyle, and medical follow-up for the management of arterial hypertension. Select the appropriate number which best describes the frequency of each behavior.

During the last month, how often did you…	Never	Rarely	Sometimes	Always	Often
1. Forget to take your medicine for arterial hypertension;	0	1	2	3	4
2. Omit to take your medicine for arterial hypertension because of its side effect;	0	1	2	3	4
3. Omit to take your medicine for arterial hypertension since you felt better;	0	1	2	3	4
4. Omit to take your medicine for arterial hypertension when you were outside (eg, travel, work);	0	1	2	3	4
5. Adapt the dose of the medicine for hypertension as your doctor suggested;	0	1	2	3	4
6. Eat fruit and vegetables;	0	1	2	3	4
7. Eat fatty foods;	0	1	2	3	4
8. Eat salty foods (eg. Tinned food, cold meat, feta);	0	1	2	3	4
9. Shake salt on your food;	0	1	2	3	4
10. Read the label of food as fir lipids and salt;	0	1	2	3	4

During the last month how often did you…	Never	Rarely	Sometimes	Always	Often
11. Try to maintain a desired body weight;	0	1	2	3	4
12. Omit to exercise as your doctor suggested;	0	1	2	3	4
13. Drink more than 2 units of alcohol (eg, whiskey, vodka) (for the men) and 1 unit (for the women) at daily basis;	0	1	2	3	4
14. Drink more than 2 glasses of wine or 2 tin of beer (for the men)/ 1 glass of wine or 1 tin of beer (for the women) at daily basis;	0	1	2	3	4
15. Smoke;	0	1	2	3	4
16. Measure your blood pressure daily;	0	1	2	3	4
During the last year, how often did you…					
17. Miss your scheduled appointment;	0	1	2	3	4
18. Miss the recommend check up;	0	1	2	3	4
